# Rates of medical service utilisation in people with and without cancer: an Australian cohort study

**DOI:** 10.1007/s00520-026-10534-z

**Published:** 2026-03-11

**Authors:** Huah Shin Ng, Elizabeth Buckley, Richard Woodman, Bogda Koczwara

**Affiliations:** 1 Flinders Health and Medical Research Institute, College of Medicine and Public Health, Flinders University, Sturt Road, Bedford Park, Adelaide, SA 5042 Australia; 2 SA Pharmacy, Northern Adelaide Local Health Network, Adelaide, South Australia Australia; 3 Australian Research Centre for Cancer Survivorship, Faculty of Medicine and Health, University of New South Wales, Sydney, New South Wales Australia

**Keywords:** Cancer, Medical service use, Health service research, Australia, Observational study

## Abstract

**Purpose:**

To explore the rates of medical service utilisation in people with and without cancer and the characteristics related to these rates.

**Methods:**

Data of respondents aged ≥ 25 years from two Australian National Health Surveys 2014–2015 and 2020–2021 were linked to medical services records from the Medicare Benefits Schedule through the Person Level Integrated Data Asset. Comparisons by cancer status and age group (< 65 years versus ≥ 65 years) were conducted using negative binomial regression, while latent class analysis (LCA) was used to explore patterns of medical service use.

**Results:**

A total of 3636 people with cancer and 18,477 people without cancer were included. Relative to younger adults without cancer, the rate of any medical services was highest in older adults with cancer (adjusted rate ratio (aRR) = 1.43; 95% CI = 1.35–1.52). This was followed by younger adults with cancer (aRR = 1.31; 95% CI = 1.25–1.38) and older adults without cancer (aRR = 1.12; 95% CI = 1.06–1.18). Characteristics associated with a higher rate of medical services use were older age, being unemployed, having polypharmacy, and a higher number of health conditions in both cancer and non-cancer groups. LCA identified three distinct patterns of medical service use including an older group of cancer survivors with a higher burden of comorbidities and polypharmacy and complex needs for multiple types of services.

**Conclusion:**

Cancer, age, multimorbidity and polypharmacy are strongly associated with medical service use. Research into predictors of health service use is crucial to inform the development of optimal approaches for care delivery, such as integrated onco-geriatric service models, tailored for populations with the highest usage.

**Supplementary Information:**

The online version contains supplementary material available at 10.1007/s00520-026-10534-z.

## Introduction

Rising costs of health care is a significant concern for the healthcare system worldwide with people with cancer likely to have increased use of health services due to treatment and supportive care needs [1–3]. An Australian study analysed self-reported data from the National Health Surveys 2011 and 2014 and showed that people with cancer were more likely to be admitted to hospital compared to people without cancer [1], but there are limited data on the use of other medical services. A systematic review of the patterns of health service use among adults with cancer included 38 studies from across 6 countries covering the United States, United Kingdom, Netherlands, Canada, France and Denmark [4]. The review identified variation in health services use between older and younger adults with cancer whereby younger people were more likely to receive preventive care, follow-up screening, visit their physicians and utilise mental health services [4]. However, the majority of the studies included in the review were from the USA, and the extent to which these findings can be generalised to other healthcare systems and populations (e.g. cancer compared to general population) remains to be explored. Understanding variation in health service use, including the predictors and types of services associated with higher use, can assist with service planning and inform the development of optimal approaches of care delivery and targeted interventions for populations who require higher use [5].

In Australia, over one-quarter (AUD$27.7 billion) of the government’s health spending in 2022–2023 was on Medicare Benefits Schedule (MBS) [6]. The MBS covers a range of medical services subsidised by the Australian Government including consultations with health practitioners (e.g. general practitioners (GP) and specialists), procedures and tests (e.g. diagnostic imaging and pathology tests) provided out-of-hospital as well as in-hospital services to private patients (e.g. with private health insurance), but does not include in-hospital services provided to public patients. While there is published data on the patterns of healthcare services before and after cancer diagnosis [7, 8], much less is known about the rate of outpatient services utilisation in people with cancer compared with those without cancer, and how this differs across age groups. The administrative data generated at every encounter with the healthcare system such as through a visit to a physician’s office, provides a unique data source to assess the patterns of service use in the real-world setting.

Our primary research question was “are there any differences in medical service use between people with and without cancer and by age groups (< 65 versus ≥ 65 years)?” We examined this by using linked administrative health and survey data to identify characteristics and patterns of service use by age and cancer status.

## Methods

### Data sources

We used data from two National Health Surveys conducted by the Australian Bureau of Statistics (ABS) in 2014–2015 via face-to-face interview and 2020–2021 via self-completed online form [9, 10]. These data comprised two independent samples from both surveys and were linked to medical services records (MBS) [11], prescription medication data (Pharmaceutical Benefits Scheme; PBS) [12] and death registry from the Australian States and Territories through the Person Level Integrated Data Asset [13]. The de-identified data were accessed through the ABS secure DataLab [14].

### Study design and population

We performed a 12-month retrospective cohort study that included all individuals aged ≥ 25 years who survived 12 months from the completion of their National Health Survey. Individuals with cancer were identified according to a self-reported history of cancer. All other participants who did not report having had a cancer were classified in the non-cancer group. Follow-up for medical service utilisation was for a 12-month period from the survey completion date.

### Outcome measures

The primary outcome of interest was the number of MBS eligible medical services used during the follow-up period. Non-Medicare services including dental services and specific item codes related to bulk billing incentives (e.g. extra payments provided to doctors who bulk bill patients from specific groups including children under 16 years and concession card holders) were excluded from the count. To avoid double counting, identical MBS item codes on the same day were considered as one medical service use.

The individual types of medical services were identified using the Medicare Broad Types of Service classification [15]. We categorised individual services into four broad categories: (1) GP (GP/vocationally registered GP, enhanced primary care, and other non-referred services), (2) specialist, (3) procedures and tests (operations, anaesthetics, pathology collection items, pathology tests, and diagnostic imaging), (4) other health professionals (practice nurse, other allied health, and optometry) and other MBS items.

### Age groupings and other variables

Survey respondent’s age was classified as < 65 (“younger”) or ≥ 65 years old (“older” Australians as defined by the Australian Institute of Health and Welfare) [16].

Additional sociodemographic data included sex, country of birth, marital status, geographical remoteness, education level, employment status, and socioeconomic status based on weekly personal income. Lifestyle behaviours and health-related factors included smoking status, fruits and vegetables consumption, alcohol intake, physical activity levels, body mass index, and number of other self-reported current health conditions (“comorbidities”) based on broad disease groupings [17].

We used Anatomical Therapeutic Chemical codes by chemical substance to identify the number of individual types of medicines received by the study population one-year prior to the National Health Survey completion date for the computation of polypharmacy (defined as having ≥ 5 medicines) using PBS data [18]. Each medication had to be dispensed at least four times during the one-year period to be included in the count of polypharmacy [18]. We categorised polypharmacy as a binary variable (yes/no).

### Statistical analysis

The study population was stratified by cancer status and age group and characterised using summary descriptive statistics. Crude rates of medical service utilisation (overall number of services and broad individual types of medical services) were calculated as the number of services per 100 person-years of follow-up. A comparison of the rates was performed using negative binomial regression between those with and without cancer and by older and younger age groups. Models were adjusted for potential confounders including sociodemographic and lifestyle factors, number of comorbidities, polypharmacy, and survey year. Results were presented as adjusted rate ratios (aRR) with 95% confidence intervals (CI).

We used multivariable negative binomial regression models to examine the characteristics associated with overall medical service utilisation and individual types of services including GP, specialist and procedures and tests, with stratification by cancer status (cancer and non-cancer groups). Variables considered were sociodemographic, lifestyle factors, number of comorbidities, polypharmacy, survey year, and, for the cancer group only, an additional variable on their disease status (current cancer or non-current).

We explored the patterns of medical services use (based on binary indicators for each individual type of service: 0–1 versus ≥ 2 visits) in the cancer and non-cancer groups using latent class analysis (LCA). A 3-class model was chosen according to the Bayesian Information Criteria statistics and model parsimony. Separate LCAs were performed according to cancer status and age group.

All the analyses were conducted using R version 4.2.1. 

## Ethics approval

This study was approved by the Flinders University Human Ethics Low Risk Panel (#7209) and was performed in accordance with the ethical standards of the 1964 Declaration of Helsinki and its later amendments.

## Results

### Characteristics of study population

A total of 3636 people with cancer and 18,477 people without cancer were analysed (Table 1). Of these, approximately half of people with cancer (*n* = 1936; 53%) and one-fifth of those without cancer (*n* = 3761; 20%) were aged ≥ 65 years. A higher proportion of people with cancer were born in Australia (78% versus 67%), separated/widowed/divorced (32% versus 22%), and unemployed (59% versus 34%); had a lower socioeconomic status (37% versus 29% in decile 1–4); and had a higher burden of comorbidities (89% versus 77% with ≥ 1 other health conditions) and polypharmacy (18% versus 7%) compared to people without cancer.

**Table 1:** Characteristics of the study cohort across two National Health Surveys by cancer status and age group

Characteristics	Overall study population, *n* (%)		Age at study entry < 65 years, *n*(%)		Age at study entry ≥ 65 years, *n*(%)	
	Non-cancer *n*=18,477	Cancer *n*=3636	Non-cancer *n*=14,716	Cancer *n*=1700	Non-cancer *n*=3761	Cancer *n*=1936
Sex						
Female	10047 (54)	2035 (56)	7952 (54)	1029 (61)	2095 (56)	1006 (52)
Male	8430 (46)	1601 (44)	6764 (46)	671 (39)	1666 (44)	930 (48)
Country of birth						
Australia	12,318 (67)	2831 (78)	10,009 (68)	1400 (82)	2309 (61)	1431 (74)
Others	6159 (33)	805 (22)	4707 (32)	300 (18)	1452 (39)	505 (26)
Geographical location						
Major cities	12,167 (66)	2203 (61)	9904 (67)	1035 (61)	2263 (60)	1168 (60)
Inner/ outer regionals/ remote	6310 (34)	1433 (39)	4812 (33)	665 (39)	1498 (40)	768 (40)
Marital status						
Married	9930 (54)	2019 (56)	7915 (54)	927 (55)	2015 (54)	1092 (56)
Never married	4452 (24)	445 (12)	4230 (29)	344 (20)	222 (6)	101 (5)
Separated/ widowed/ divorced	4095 (22)	1172 (32)	2571 (17)	429 (25)	1524 (40)	743 (38)
Education level						
Postgraduate	2299 (12)	393 (11)	2041 (14)	247 (14)	258 (7)	146 (8)
Bachelor	4070 (22)	629 (17)	3619 (25)	317 (19)	451 (12)	312 (16)
Diploma	2115 (11)	432 (12)	1811 (12)	226 (13)	304 (8)	206 (11)
Certificate	3674 (20)	732 (20)	3024 (20)	385 (23)	650 (17)	347 (18)
No non-school qualification	5961 (32)	1355 (37)	3967 (27)	494 (29)	1994 (53)	861 (44)
Not known	358 (2)	95 (3)	254 (2)	31 (2)	104 (3)	64 (3)
Employment status						
Employed	12,229 (66)	1479 (41)	11,590 (79)	1208 (71)	639 (17)	271 (14)
Unemployed/not in labour force	6248 (34)	2157 (59)	3126 (21)	492 (29)	3122 (83)	1665 (86)
Equivalised personal weekly income						
Decile 1–2 (most disadvantaged)	2149 (12)	361 (10)	1799 (12)	213 (13)	350 (9)	148 (8)
Decile 3–4	3241 (17)	1000 (27)	1606 (11)	244 (14)	1635 (43)	756 (39)
Decile 5–6	3450 (19)	787 (22)	2629 (18)	284 (17)	821 (22)	503 (26)
Decile 7–8	3892 (21)	616 (17)	3523 (24)	377 (22)	369 (10)	239 (12)
Decile 9–10	4414 (24)	618 (17)	4173 (28)	469 (28)	241 (6)	149 (8)
Not known	1331 (7)	254 (7)	986 (7)	113 (7)	345 (10)	141 (7)
Body mass index						
Normal/underweight	6512 (35)	1163 (32)	5349 (36)	559 (33)	1163 (31)	604 (31)
Overweight	6614 (36)	1342 (37)	5163 (35)	587 (35)	1451 (39)	755 (39)
Obese	5085 (28)	1089 (30)	3980 (27)	531 (31)	1105 (29)	558 (29)
Missing	266 (1)	42 (1)	224 (2)	23 (1)	42 (1)	19 (1)
Smoking status						
Never smoked	9978 (54)	1775 (49)	8020 (55)	795 (47)	1958 (52)	980 (51)
Ex-smoker	5991 (32)	1519 (42)	4424 (30)	664 (39)	1567 (42)	855 (44)
Current smoker	2508 (14)	342 (9)	2272 (15)	241 (14)	236 (6)	101 (5)
Met recommended vegetable/ fruits guidelines						
Met both	1111 (6)	286 (8)	818 (6)	116 (7)	293 (8)	170 (9)
Met either	8240 (45)	1802 (50)	6257 (42)	755 (44)	1983 (53)	1047 (54)
Not met/ missing	9126 (49)	1548 (43)	7641 (52)	829 (49)	1485 (39)	719 (37)
Alcohol intake						
Not applicable/ not known	3819 (21)	736 (20)	2774 (19)	277 (16)	1045 (28)	459 (24)
Everyday	1067 (6)	373 (10)	641 (4)	116 (7)	426 (11)	257 (13)
2–6 days a week	7544 (41)	1518 (42)	6243 (42)	778 (46)	1301 (35)	740 (38)
1–3 days a month	3240 (18)	547 (15)	2800 (19)	302 (18)	440 (12)	245 (13)
Less than once a month	2807 (15)	462 (13)	2258 (15)	227 (13)	549 (15)	235 (12)
Met physical activity guidelines						
Yes	3739 (20)	906 (25)	2644 (18)	325 (19)	1095 (29)	581 (30)
No	14,738 (80)	2730 (75)	12,072 (82)	1375 (81)	2666 (71)	1355 (70)
Number of health conditions (excluding cancer)						
0	4308 (23)	398 (11)	3930 (27)	259 (15)	378 (10)	139 (7)
1–2	9225 (50)	1609 (44)	7534 (51)	813 (48)	1691 (45)	796 (41)
3–4	3974 (22)	1221 (34)	2669 (18)	467 (27)	1305 (35)	754 (39)
≥ 5	970 (5)	408 (11)	583 (4)	161 (9)	387 (10)	247 (13)
Polypharmacy						
No	17,102 (93)	2971 (82)	14256 (97)	1566 (92)	2846 (76)	1405 (73)
Yes	1375 (7)	665 (18)	460 (3)	134 (8)	915 (24)	531 (27)
Cancer status						
Current cancer	NA	534 (15)	N/A	220 (13)	N/A	314 (16)
Non-current		3102 (85)		1480 (87)		1622 (84)

Key: *NA* not applicable

There were differences by age group regardless of cancer status whereby a higher proportion of people in the older age group had a lower education level, had a lower socioeconomic status, were being unemployed, and had a higher burden of comorbidities and polypharmacy than the younger age group counterparts.

### Medical service utilisation

The crude rate of any medical service use was higher in older adults with cancer (3984/100 person-years), when compared to older adults without cancer (2988/100 person-years), younger adults with cancer (2484/100 person-years) and younger adults without cancer (1555/100 person-years) (Supplementary Fig. 1). When examining types of medical services separately, older adults with cancer had the highest crude rate across four broad types of medical services including GP, specialist, procedures and tests, and other health professionals/other MBS items, followed by older adults without cancer, younger adults with cancer, and younger adults without cancer.

When compared to younger adults without cancer, the relative rate of any medical service utilisation was highest in older adults with cancer (aRR = 1.43; 95% CI = 1.35–1.52), followed by younger adults with cancer (aRR = 1.31; 95% CI = 1.25–1.38) and older adults without cancer (aRR = 1.12; 95% CI = 1.06–1.18; Table 2). Similar trends were observed for GP services and procedures and tests.

**Table 2:** Medical service use by cancer status and age groups across two National Health Surveys

Medical service use by cancer status and age group	Adjusted rate ratio^a^ (95% CI)	*p*-value
Any medical services		
Non-cancer & younger	Reference	< 0.001
Cancer & older	1.43 (1.35–1.52)*	
Cancer & younger	1.31 (1.25–1.38)*	
Non-cancer & older	1.12 (1.06–1.18)*	
By types of services:		
(i) General practitioner including non-referred: GP/VRGP, enhanced primary care and other		
Non-cancer & younger	Reference	< 0.001
Cancer & older	1.21 (1.15–1.27)*	
Cancer & younger	1.12 (1.08–1.17)*	
Non-cancer & older	1.11 (1.06–1.16)*	
(ii) Specialist attendance		
Non-cancer & younger	Reference	< 0.001
Cancer & older	1.59 (1.41–1.79)*	
Cancer & younger	1.68 (1.53–1.85)*	
Non-cancer & older	1.09 (0.98–1.21)	
(iii) Procedures and tests including operations, anaesthetics, pathology collection items, pathology tests, and diagnostic imaging		
Non-cancer & younger	Reference	< 0.001
Cancer & older	1.53 (1.41–1.65)*	
Cancer & younger	1.39 (1.30–1.48)*	
Non-cancer & older	1.13 (1.06–1.21)*	
(iv) Other health professionals including practice nurse items, other allied health, optometry and other MBS items		
Non-cancer & younger	Reference	< 0.001
Cancer & older	1.25 (1.14–1.38)*	
Cancer & younger	1.28 (1.19–1.38)*	
Non-cancer & older	1.10 (1.01–1.20)*	

Key: *GP* general practitioner; *MBS* Medicare Benefits Schedule; *VRGP* vocationally registered general practitioner

^a^The negative binomial regression model was adjusted for sex, age, country of birth, geographical location, marital status, education level, employment status, socioeconomic status, body mass index, smoking status, vegetable/fruit intake, alcohol intake, physical activity, number of health conditions (excluding cancer), polypharmacy status and survey years

*p-value < 0.05

For specialist services, the relative rate of specialist attendance was highest in younger adults with cancer (aRR = 1.68; 95% CI = 1.53–1.85), followed by older adults with cancer (aRR = 1.59; 95% CI = 1.41–1.79), and older adults without cancer (aRR = 1.09; 95% CI = 0.98–1.21) when compared to younger adults without cancer. The relative rates of other health professionals/other MBS items followed a similar trend.

### Characteristics associated with medical service use

Several characteristics including older age, being unemployed, completing the survey in a more recent year, polypharmacy, and a higher number of health conditions were associated with higher rates of any medical service use in both cancer and non-cancer groups (Table 3). Similar trends were observed for individual types of services including GP, specialist, and procedures and tests. Having resided outside of major cities and being a current smoker were associated with a lower rate of any medical service use in both cancer and non-cancer groups.

**Table 3:** Multivariable models assessing the characteristics associated with medical service utilisation by cancer status

Characteristics	Adjusted rate ratio (95% confidence interval)							
	Any medical services		General practitioner		Specialist		Procedures and tests	
	Non-cancer	Cancer	Non-cancer	Cancer	Non-cancer	Cancer	Non-cancer	Cancer
Sex								
Female	Reference	Reference	Reference	Reference	Reference	Reference	Reference	Reference
Male	0.74 (0.72–0.76)*	0.96 (0.90–1.02)	0.79 (0.77–0.82)*	0.92 (0.87–0.97)*	0.83 (0.77–0.88)*	0.97 (0.87–1.08)	0.71 (0.69–0.74)*	1.00 (0.92–1.07)
Age group								
< 65 years	Reference	Reference	Reference	Reference	Reference	Reference	Reference	Reference
≥ 65 years	1.35 (1.29–1.41)*	1.34 (1.25–1.44)*	1.22 (1.18–1.27)*	1.24 (1.16–1.32)*	1.79 (1.63–1.97)*	1.47 (1.29–1.66)*	1.38 (1.30–1.46)*	1.37 (1.26–1.50)*
Country of birth								
Australia	Reference	Reference	Reference	Reference	Reference	Reference	Reference	Reference
Others	0.98 (0.95–1.01)	1.00 (0.94–1.07)	1.05 (1.02–1.08)*	1.05 (0.99–1.11)	0.76 (0.71–0.82)*	0.92 (0.82–1.03)	0.97 (0.93–1.01)	0.99 (0.91–1.07)
Geographical location								
Major cities	Reference	Reference	Reference	Reference	Reference	Reference	Reference	Reference
Inner/ outer regionals/ remote	0.86 (0.84–0.89)*	0.81 (0.77–0.86)*	0.89 (0.87–0.92)*	0.93 (0.88–0.97)*	0.82 (0.77–0.88)*	0.69 (0.62–0.76)*	0.85 (0.82–0.88)*	0.78 (0.73–0.83)*
Marital status								
Married	Reference	Reference	Reference	Reference	Reference	Reference	Reference	Reference
Never married	0.84 (0.81–0.87)*	1.00 (0.91–1.09)	0.92 (0.89–0.95)*	1.05 (0.97–1.13)	0.74 (0.69–0.80)*	0.94 (0.80–1.10)	0.80 (0.77–0.84)*	0.98 (0.88–1.09)
Separated/ widowed/ divorced	0.99 (0.96–1.03)	0.98 (0.92–1.04)	1.04 (1.01–1.07)*	1.04 (0.98–1.09)	0.97 (0.90–1.05)	0.88 (0.79–0.98)*	0.96 (0.91–1.00)	0.97 (0.90–1.04)
*P*-value	< 0.001	0.818	< 0.001	0.304	< 0.001	0.074	< 0.001	0.656
Education level								
Postgraduate	Reference	Reference	Reference	Reference	Reference	Reference	Reference	Reference
Bachelor	1.01 (0.96–1.06)	0.93 (0.83–1.03)	1.02 (0.98–1.07)	0.93 (0.85–1.02)	1.02 (0.92–1.14)	0.98 (0.81–1.18)	1.01 (0.95–1.08)	0.91 (0.80–1.04)
Diploma	1.03 (0.97–1.10)	1.09 (0.97–1.22)	1.11 (1.06–1.17)*	1.06 (0.96–1.17)	1.00 (0.88–1.14)	0.99 (0.81–1.22)	1.01 (0.94–1.09)	1.09 (0.94–1.25)
Certificate	1.04 (0.99–1.10)	0.99 (0.89–1.11)	1.16 (1.11–1.22)*	1.02 (0.93–1.12)	0.92 (0.82–1.04)	0.87 (0.72–1.06)	1.01 (0.94–1.08)	1.01 (0.88–1.15)
No non-school qualification	0.99 (0.94–1.04)	1.01 (0.91–1.12)	1.13 (1.08–1.18)*	1.11 (1.02–1.21)*	0.87 (0.78–0.97)*	0.91 (0.76–1.09)	0.95 (0.89–1.02)	0.98 (0.87–1.11)
Not known	0.98 (0.88–1.09)	1.21 (1.01–1.46)*	1.06 (0.96–1.17)	1.05 (0.90–1.24)	0.98 (0.78–1.25)	1.18 (0.86–1.65)	0.96 (0.83–1.10)	1.25 (1.00–1.57)
*P*-value	0.165	0.010	< 0.001	< 0.001	0.011	0.321	0.204	0.021
Employment status								
Employed	Reference	Reference	Reference	Reference	Reference	Reference	Reference	Reference
Unemployed/not in labour force	1.13 (1.08–1.18)*	1.12 (1.04–1.21)*	1.14 (1.10–1.19)*	1.10 (1.03–1.18)*	1.21 (1.10–1.32)*	1.32 (1.16–1.50)*	1.09 (1.03–1.15)*	1.06 (0.97–1.16)
Equivalised personal weekly income								
Decile 1–2 (most disadvantaged)	1.01 (0.96–1.08)	1.07 (0.95–1.21)	1.08 (1.03–1.14)*	1.07 (0.96–1.18)	0.91 (0.80–1.04)	0.88 (0.71–1.08)	0.99 (0.92–1.07)	1.12 (0.97–1.30)
Decile 3–4	1.09 (1.03–1.15)*	1.00 (0.90–1.10)	1.24 (1.18–1.31)*	1.16 (1.07–1.27)*	0.86 (0.76–0.96)*	0.74 (0.62–0.88)*	1.01 (0.94–1.08)	0.95 (0.84–1.07)
Decile 5–6	1.05 (1.00–1.10)	1.10 (1.00–1.22)	1.16 (1.12–1.21)*	1.09 (1.00–1.18)	0.82 (0.74–0.91)*	0.92 (0.77–1.09)	1.00 (0.94–1.06)	1.12 (1.00–1.26)
Decile 7–8	1.02 (0.98–1.07)	1.04 (0.94–1.14)	1.12 (1.07–1.16)*	1.02 (0.94–1.11)	0.86 (0.79–0.95)*	1.01 (0.85–1.20)	0.99 (0.94–1.05)	1.06 (0.95–1.20)
Decile 9–10	Reference	Reference	Reference	Reference	Reference	Reference	Reference	Reference
Not known	1.12 (1.05–1.19)*	1.03 (0.91–1.17)	1.15 (1.09–1.21)*	1.15 (1.03–1.29)*	1.12 (0.98–1.28)	0.89 (0.71–1.12)	1.10 (1.01–1.19)*	1.00 (0.85–1.17)
*P*-value	0.003	0.133	< 0.001	0.004	< 0.001	0.002	0.207	0.007
Body mass index								
Normal/underweight	Reference	Reference	Reference	Reference	Reference	Reference	Reference	Reference
Overweight	0.97 (0.94–1.01)	0.99 (0.93–1.06)	1.01 (0.98–1.04)	1.02 (0.96–1.08)	0.98 (0.91–1.06)	0.99 (0.88–1.11)	0.96 (0.92–1.00)	0.99 (0.91–1.07)
Obese	1.03 (0.99–1.07)	1.01 (0.94–1.08)	1.08 (1.04–1.11)*	1.06 (1.00–1.13)	1.00 (0.92–1.08)	0.91 (0.80–1.03)	1.01 (0.96–1.06)	0.99 (0.90–1.07)
Missing	1.23 (1.09–1.39)*	0.84 (0.66–1.10)	1.14 (1.03–1.27)*	0.99 (0.80–1.23)	1.10 (0.85–1.44)	0.69 (0.44–1.13)	1.19 (1.02–1.39)*	0.80 (0.59–1.10)
*P*-value	< 0.001	0.628	< 0.001	0.276	0.840	0.203	0.007	0.580
Smoking status								
Never smoked	Reference	Reference	Reference	Reference	Reference	Reference	Reference	Reference
Ex-smoker	1.07 (1.04–1.11)*	0.95 (0.90–1.01)	1.06 (1.03–1.09)*	1.03 (0.98–1.09)	1.06 (0.99–1.13)	0.88 (0.79–0.97)*	1.07 (1.03–1.12)*	0.93 (0.86–1.00)
Current smoker	0.92 (0.88–0.97)*	0.80 (0.72–0.89)*	1.04 (1.00–1.08)*	1.04 (0.96–1.14)	0.73 (0.66–0.81)*	0.62 (0.52–0.75)*	0.87 (0.82–0.92)*	0.70 (0.62–0.80)*
*P*-value	< 0.001	< 0.001	< 0.001	0.376	< 0.001	< 0.001	< 0.001	< 0.001
Met recommended vegetable/ fruits guidelines								
Met both	Reference	Reference	Reference	Reference	Reference	Reference	Reference	Reference
Met either	0.98 (0.92–1.04)	0.97 (0.87–1.07)	1.03 (0.97–1.08)	1.01 (0.93–1.11)	0.96 (0.84–1.10)	0.91 (0.75–1.09)	0.99 (0.92–1.07)	0.96 (0.85–1.09)
Not met/ missing	0.93 (0.87–0.99)*	0.96 (0.86–1.06)	1.00 (0.95–1.05)	1.02 (0.94–1.12)	0.91 (0.79–1.03)	0.89 (0.73–1.07)	0.92 (0.85–1.00)	0.94 (0.82–1.07)
*P*-value	< 0.001	0.733	0.087	0.851	0.111	0.441	< 0.001	0.572
Alcohol intake								
Not applicable/ not known	Reference	Reference	Reference	Reference	Reference	Reference	Reference	Reference
Everyday	0.96 (0.89–1.02)	0.92 (0.83–1.02)	0.87 (0.82–0.92)*	0.89 (0.81–0.97)*	1.25 (1.08–1.45)*	0.95 (0.79–1.15)	0.98 (0.89–1.07)	0.94 (0.83–1.07)
2–6 days a week	0.89 (0.85–0.92)*	0.88 (0.82–0.95)*	0.85 (0.82–0.88)*	0.92 (0.87–0.99)*	1.05 (0.96–1.14)	0.96 (0.84–1.10)	0.91 (0.86–0.95)*	0.87 (0.80–0.96)*
1–3 days a month	0.93 (0.88–0.97)*	0.97 (0.88–1.06)	0.88 (0.85–0.92)*	0.98 (0.91–1.06)	1.06 (0.96–1.17)	0.97 (0.82–1.14)	0.95 (0.89–1.01)	0.94 (0.84–1.06)
Less than once a month	1.00 (0.95–1.05)	1.04 (0.95–1.15)	0.97 (0.93–1.01)	1.01 (0.94–1.10)	1.21 (1.09–1.35)*	1.14 (0.96–1.35)	1.01 (0.95–1.07)	1.03 (0.91–1.16)
*P*-value	< 0.001	< 0.001	< 0.001	0.011	< 0.001	0.249	< 0.001	0.012
Met physical activity guidelines								
Yes	Reference	Reference	Reference	Reference	Reference	Reference	Reference	Reference
No	1.02 (0.98–1.06)	1.12 (1.05–1.19)*	1.02 (0.99–1.05)	1.03 (0.98–1.09)	1.02 (0.94–1.11)	1.11 (0.99–1.25)	1.02 (0.97–1.07)	1.17 (1.08–1.27)*
Number of health conditions (excluding cancer)								
0	Reference	Reference	Reference	Reference	Reference	Reference	Reference	Reference
1–2	1.42 (1.37–1.47)*	1.11 (1.01–1.22)*	1.38 (1.34–1.43)*	1.25 (1.15–1.35)*	1.68 (1.55–1.83)*	0.98 (0.83–1.16)	1.37 (1.31–1.44)*	1.05 (0.94–1.18)
3–4	1.85 (1.77–1.93)*	1.35 (1.23–1.49)*	1.77 (1.70–1.84)*	1.48 (1.36–1.61)*	2.39 (2.17–2.64)*	1.43 (1.20–1.71)*	1.77 (1.67–1.88)*	1.26 (1.11–1.41)*
≥ 5	2.16 (2.01–2.32)*	1.47 (1.30–1.66)*	2.04 (1.92–2.17)*	1.64 (1.48–1.82)*	2.86 (2.47–3.33)*	1.58 (1.27–1.96)*	2.08 (1.90–2.28)*	1.36 (1.17–1.57)*
*P*-value	< 0.001	< 0.001	< 0.001	< 0.001	< 0.001	< 0.001	< 0.001	< 0.001
Survey years								
2014–15	Reference	Reference	Reference	Reference	Reference	Reference	Reference	Reference
2020–21	1.32 (1.28–1.37)*	1.23 (1.16–1.31)*	1.27 (1.24–1.31)*	1.30 (1.24–1.37)*	1.20 (1.12–1.28)*	1.06 (0.96–1.18)	1.40 (1.35–1.46)*	1.22 (1.13–1.31)*
Polypharmacy								
No	Reference	Reference	Reference	Reference	Reference	Reference	Reference	Reference
Yes	1.74 (1.65–1.85)*	1.50 (1.39–1.62)*	1.60 (1.53–1.68)*	1.45 (1.37–1.55)*	2.28 (2.03–2.56)*	1.62 (1.42–1.84)*	1.75 (1.63–1.89)*	1.52 (1.39–1.67)*
Cancer status								
Non–current	N/A	Reference	N/A	Reference	N/A	Reference	N/A	Reference
Current cancer		1.58 (1.46–1.70)*		1.12 (1.05–1.19)*		1.96 (1.72–2.24)*		1.69 (1.54–1.85)*

*N/A* not applicable. *p-value < 0.05

### Patterns of medical services identified using LCA

We identified three distinct patterns of medical services in the cancer group using LCA: pattern 1 was generally low medical service users (*n* = 971; 27%), pattern 2 was those using predominately pathology tests (*n* = 1618; 44%), and pattern 3 was high medical service users (*n* = 1047; 29%) (Supplementary Table 1). Similarly, the same three distinct patterns of medical services were identified in the non-cancer group but with a higher proportion of subjects in pattern 1 (low users, *n* = 7068; 38%) and a lower proportion of subjects in pattern 3 (high users, *n* = 3243; 18%) compared to the distribution within the cancer group.

**Fig. 1:**
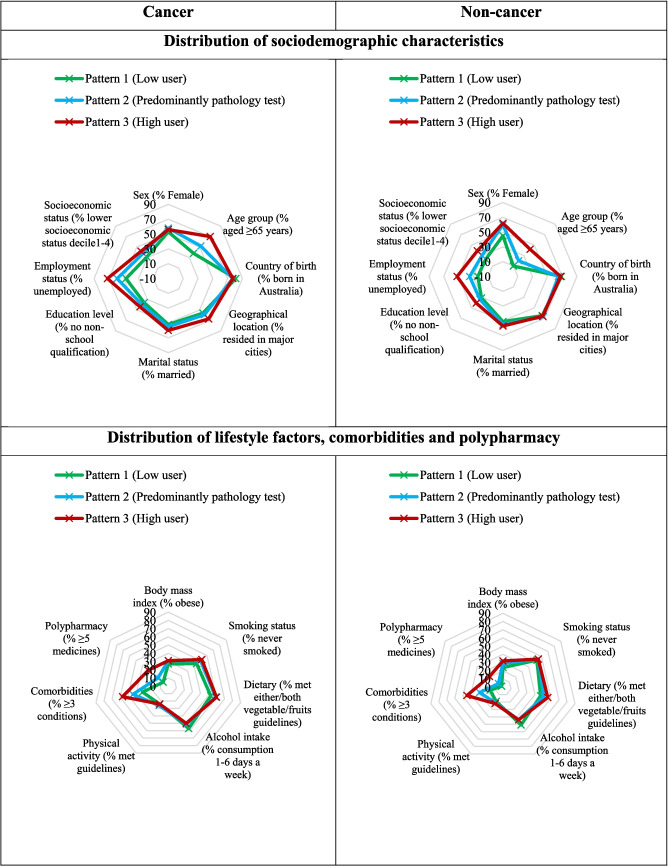
The prevalence distribution of the characteristics of people with and without cancer by patterns of medical service use identified using latent class analysis. The percentage (%) of the characteristics in each pattern is shown

Subjects in pattern 3 (high medical service users) were older, unemployed, had a lower education level and socioeconomic status, and a higher number of comorbidities and polypharmacy compared to the other two patterns in both the cancer and non-cancer groups, although these prevalences were generally higher in the cancer group (Fig. 1 and Supplementary Table 1).

When examining separately by cancer status and age group (Supplementary Table 2), the proportion of subjects with high medical service use (pattern 3) was higher in those with cancer than those without cancer in both age groups (28% with cancer versus 13% without cancer in the younger age group and 35% versus 32% in the older age group). The distribution of sociodemographic and lifestyle characteristics of high medical service users was relatively similar between people with and without cancer in their respective age groups, although the percentage of those with a higher number of comorbidities and polypharmacy tended to be higher in the cancer group (Supplementary Table 2 and Supplementary Figs. 2 & 3).

## Discussion

This large retrospective 12-month cohort study demonstrates that Australians with cancer used more medical services than those without cancer, and that the pattern of service use also differs according to age. Specifically, older adults with cancer used any medical services, GP services, and procedures and tests significantly more often than younger adults without cancer, whereas younger adults with cancer have higher specialist attendance and use other healthcare professionals/MBS service items more often than their younger counterparts without cancer. Certain characteristics, including older age, unemployment, more recent survey years, polypharmacy, and a higher number of health conditions, are associated with higher use of medical services in both cancer and non-cancer groups. Three distinct patterns of medical service use were identified by using LCA, with a ‘higher-user’ pattern which made up of nearly one-third of people with cancer and one-fifth of people without cancer showing complex needs for multiple types of services. These high users of medical services tended to be older, unemployed, have a lower education level and socioeconomic status, and a higher burden of comorbidities and polypharmacy. Our findings support the needs of developing strategies to facilitate screening and management of these risk factors and comorbidities among individuals with high medical service use.

Older adults with cancer had the highest rate of any medical service utilisation, followed by younger adults with cancer and older adults without cancer, when compared to younger adults without cancer. A higher medical service utilisation may reflect greater clinical complexity and supportive care needs of people with cancer. Older adults with cancer often have multiple chronic diseases and other age-related conditions such as functional and cognitive impairment [19–21]. In recognising the specific needs of older adults with cancer, geriatric oncology specialty has emerged to address and improve the health outcomes of this vulnerable population group. However, geriatric oncology remains a niche service that is often restricted to tertiary academic hospitals [22]. A prior study conducted in the United States showed that older cancer survivors spend about one month per calendar year receiving care in the ambulatory (outpatient) setting such as visits to clinicians, procedures, imaging and tests, which are often delivered by non-oncology specialties [23]. The findings have important implications for the cancer care delivery in older cancer survivors such as the implementation of an integrated care model [24] to lessen the burden of cancer and other chronic diseases on the health system and to also address the likely greater clinical complexity and supportive care needs of this population, while recognising workforce challenges in primary care, geriatrics and oncology [25, 26]. Our study also shows different needs for medical services in younger adults. Specifically, younger adults with cancer had higher rates for specialist attendance compared to younger adults without cancer, followed by older adults with cancer and older adults without cancer. As cancer incidence in younger people continues to rise [27], this will be an additional area of burden on healthcare services.

Several factors associated with higher medical service use are older age, unemployment, polypharmacy, and a higher number of comorbidities. By contrast, two other factors linked to lower medical service use were having resided outside of major cities and being a current smoker. These risk factors appeared to be similar in both cancer and non-cancer groups and across different types of medical services. While other studies have consistently demonstrated the association between polypharmacy and comorbidity and greater healthcare utilisation including hospital and emergency department visits [28–31], our study expanded the findings by examining the broader medical service use. Geographical remoteness also plays a part whereby people living in inner/outer regionals and remote areas have a lower rate of medical service use which may be due to poorer accessibility of health services and higher rates of behaviours associated with chronic conditions such as smoking in these regions compared to major cities [32]. Our findings further support the needs of developing a strategic approach to maximise the provision of effective health services in rural/remote areas to minimise the disparity between rural and urban’s population health outcomes [33]. Visits to doctors in the outpatient or primary care settings are important to increase the uptake of preventive services such as tobacco use screening and counselling [34, 35]. However, given lower rates of medical services among current smokers, personalised and interactive online-based smoking cessation interventions [36] may offer an alternative strategy aiming at this vulnerable group of people.

By using the LCA, we identified a specific group of users among those with cancer that tended to be older, were unemployed, and had a lower education level and socioeconomic status, a higher burden of comorbidities, and polypharmacy. This group showed complex needs for multiple types of services. The proportion of those with high medical service use was higher in people with cancer than their counterparts without cancer in both age groups (< 65 and ≥ 65 years). Our findings highlight the importance of an integrated care model that offers flexibility in delivery aiming to provide a coordinated, multidisciplinary approach to improve health outcomes and patient experiences especially in older adults with cancer with a high burden of comorbidity, polypharmacy, and complex needs of health services [24]. Whether these increased needs for medical services were due to cancer or comorbidity or both conditions require further exploration.

This study has several limitations. While we were able to minimise potential recall bias by accessing the MBS data to examine patterns of medical services use, other health information such as cancer status and lifestyle factors were self-reported which may introduce response bias [37]. Our study excluded individuals who died within 12 months of survey completion due to the very different healthcare patterns that occur at the end-of-life care. Data on cancer stage, types, and diagnosis date were not available, and therefore, we were unable to perform further subgroup analysis. Thus, people with cancer in our study cohort likely represent a heterogenous mixture of individuals across active treatment and survivorship phases. Although we classified survey respondents’ age into two groups using a cut-off of 65 years which is a common threshold used to define older adult populations, we cannot rule out the potential for residual age-related heterogeneity within these broad age groups. Even though we adjusted for survey year in the analysis, we were not able to account for other factors such as changes in healthcare delivery (e.g. expansion of telehealth and deferred care during the COVID-19 pandemic when the National Health Survey 2020–2021 was conducted), and the evolution of cancer treatment over the study period that may have influenced the health utilisation patterns. While the MBS data captured a broad range of services delivered by health practitioners, a proportion of allied health services were not covered by Medicare (e.g. paying for the service completely out-of-pocket) which may result in underestimation of those services [38]. The MBS only subsidises (and therefore captures) part of the total healthcare expenditure in Australia, and other major health spending areas include hospitalisations and pharmaceuticals subsidised by the Australian Government were not examined in this study. Nonetheless, we have reported data on hospitalisations in people with cancer compared to the general population in a previous study [1], and the current study provides further insights into medical services use to inform health service planning at the systems level. It is possible that some medications received by people with cancer could include components of cancer treatment and supportive therapy in the computation of polypharmacy using PBS data. Future research should examine the characteristics of people who persist in their high utilisation of health services over a longer duration and the types of individual comorbidities and cancer treatment associated with higher use. Effective prediction of high medical service users would help target interventions, given that the existing supportive interventions developed for older people with cancer often did not address complex issues including multimorbidity and other geriatric syndromes (e.g. polypharmacy) in addition to cancer [39].

## Conclusion

Cancer, age, multimorbidity, and polypharmacy are strongly associated with medical service use. Research into predictors of health service use is crucial to inform the development of optimal approaches for care delivery, such as integrated onco-geriatric service models, tailored for this population with the highest usage.

## Supplementary Information

Below is the link to the electronic supplementary material.

ESM 1 (DOCX 77.3 KB)

## Data Availability

The data may be accessed through the Australian Bureau of Statistics with the appropriate approvals.

## References

[1] Ng HS, Koczwara B, Roder D et al (2020) Patterns of health service utilisation among the Australian population with cancer compared with the general population. Aust Health Rev 44(3):470–9. 10.1071/AH18184 31693479 10.1071/AH18184

[2] Mols F, Helfenrath KA, Vingerhoets AJJM et al (2007) Increased health care utilization among long-term cancer survivors compared to the average Dutch population: a population-based study. Int J Cancer 121(4):871–7. 10.1002/ijc.22739 17417782 10.1002/ijc.22739

[3] Hanchate AD, Clough-Gorr KM, Ash AS et al (2010) Longitudinal patterns in survival, comorbidity, healthcare utilization and quality of care among older women following breast cancer diagnosis. J Gen Intern Med 25(10):1045–50. 10.1007/s11606-010-1407-9 20532657 10.1007/s11606-010-1407-9 PMC2955471

[4] Treanor C, Donnelly M (2012) An international review of the patterns and determinants of health service utilisation by adult cancer survivors. BMC Health Serv Res 12:316. 10.1186/1472-6963-12-316 22973899 10.1186/1472-6963-12-316 PMC3465193

[5] Morris JN, Crawford-Williams F, Koczwara B et al (2023) Current landscape of cancer survivorship research in Australia. Asia Pac J Clin Oncol 19(5):e305–e13. 10.1111/ajco.13914 36658677 10.1111/ajco.13914

[6] Australian Institute of Health and Welfare (AIHW). Health expenditure Australia 2022–23 2024 [Available from: https://www.aihw.gov.au/reports/health-welfare-expenditure/health-expenditure-australia-2022-23/contents/spending-trends-by-source/government-sources-australian-government-spending].

[7] Morrell S, Young J, Roder D (2019) The burden of cancer on primary and secondary health care services before and after cancer diagnosis in New South Wales, Australia. BMC Health Serv Res 19(1):431. 10.1186/s12913-019-4280-1 31248405 10.1186/s12913-019-4280-1 PMC6598375

[8] Christensen KG, Fenger-Grøn M, Flarup KR, Vedsted P (2012) Use of general practice, diagnostic investigations and hospital services before and after cancer diagnosis - a population-based nationwide registry study of 127,000 incident adult cancer patients. BMC Health Serv Res 12(1):224. 10.1186/1472-6963-12-224 22838741 10.1186/1472-6963-12-224 PMC3507912

[9] Australian Bureau of Statistics. National Health Survey: first results methodology: Australian Bureau of Statistics; 2022 [Available from: https://www.abs.gov.au/methodologies/national-health-survey-first-results-methodology/2020-21.].

[10] Australian Bureau of Statistics. 4364.0.55.001 - National Health Survey: first results, 2014–15 2015 [Available from: https://www.abs.gov.au/AUSSTATS/abs@.nsf/Lookup/4364.0.55.001Explanatory%20Notes12014-15?OpenDocument].

[11] Australian Government Department of Health and Aged Care. Medicare Benefits Schedule 2024 [Available from: https://www.mbsonline.gov.au/].

[12] Australian Government Department of Health and Aged Care. The Pharmaceutical Benefits Scheme (PBS) 2024 [Available from: https://www.pbs.gov.au/pbs/home].

[13] Australian Bureau of Statistics. Microdata: Person Level Integrated Data Asset (PLIDA) 2024 [Available from: https://www.abs.gov.au/statistics/microdata-tablebuilder/available-microdata-tablebuilder/person-level-integrated-data-asset-plida].

[14] Australian Bureau of Statistics. DataLab: Australian Bureau of Statistics; 2021 [Available from: https://www.abs.gov.au/statistics/microdata-tablebuilder/datalab].

[15] Australian Institute of Health and Welfare (AIHW). Record—Department of Health Broad Type of Service (BTOS) hierarchy, code NNNN: AIHW; 2015 [Available from: http://meteor.aihw.gov.au/content/index.phtml/itemId/604330].

[16] Australian Institute of Health and Welfare (AIHW). Older Australians 2024 [Available from: https://www.aihw.gov.au/reports/older-people/older-australians/contents/summary].

[17] Ng HS, Roder D, Koczwara B, Vitry A (2018) Comorbidity, physical and mental health among cancer patients and survivors: an Australian population-based study. Asia Pac J Clin Oncol 14(2):e181–e192. 10.1111/ajco.12677 28371441 10.1111/ajco.12677

[18] Australian Commission on Safety and Quality in Health Care. The Fourth Atlas of Healthcare Variation 2020 - Medicines use in older people - full chapter 2021 [Available from: https://www.safetyandquality.gov.au/publications-and-resources/resource-library/fourth-atlas-healthcare-variation-2020-medicines-use-older-people-full-chapter].

[19] Bluethmann SM, Mariotto AB, Rowland JH (2016) Anticipating the “silver tsunami”: prevalence trajectories and co-morbidity burden among older cancer survivors in the United States. Cancer Epidemiol Biomarkers Prev 25(7):1029–1036. 10.1158/1055-9965.Epi-16-0133 27371756 10.1158/1055-9965.EPI-16-0133 PMC4933329

[20] Magnuson A, Ahles T, Chen BT et al (2021) Cognitive function in older adults with cancer: assessment, management, and research opportunities. J Clin Oncol 39(19):2138–49. 10.1200/jco.21.00239 34043437 10.1200/JCO.21.00239 PMC8260910

[21] Kleckner AS, Magnuson A (2022) The nutritional needs of older cancer survivors. J Geriatr Oncol 13(5):738–741. 10.1016/j.jgo.2021.12.007 34906443 10.1016/j.jgo.2021.12.007 PMC9187777

[22] Soto-Perez-de-Celis E, de Glas NA, Hsu T et al (2017) Global geriatric oncology: achievements and challenges. J Geriatr Oncol 8(5):374–86. 10.1016/j.jgo.2017.06.001 28642040 10.1016/j.jgo.2017.06.001

[23] Gupta A, Chant ED, Mohile S et al (2024) Health care contact days among older cancer survivors. JCO Oncol Pract 20(7):943–52. 10.1200/op.23.00590 38452315 10.1200/OP.23.00590 PMC11268556

[24] Soo WK, Yin V, Crowe J et al (2023) Integrated care for older people with cancer: a primary care focus. Lancet Healthy Longev 4(6):e243–e5. 10.1016/s2666-7568(23)00058-2 37172606 10.1016/S2666-7568(23)00058-2

[25] Jefford M, Howell D, Li Q et al (2022) Improved models of care for cancer survivors. Lancet 399(10334):1551–60. 10.1016/s0140-6736(22)00306-3 35430022 10.1016/S0140-6736(22)00306-3 PMC9009839

[26] Kent EE, Park EM, Wood WA et al (2021) Survivorship care of older adults with cancer: priority areas for clinical practice, training, research, and policy. J Clin Oncol 39(19):2175–84. 10.1200/jco.21.00226 34043450 10.1200/JCO.21.00226 PMC8260922

[27] Australian INstitute of Health and Welfare (AIHW). Cancer data in Australia 2024 [Available from: https://www.aihw.gov.au/reports/cancer/cancer-data-in-australia/contents/overview].

[28] Fried TR, O’Leary J, Towle V et al (2014) Health outcomes associated with polypharmacy in community-dwelling older adults: a systematic review. J Am Geriatr Soc 62(12):2261–72. 10.1111/jgs.13153 25516023 10.1111/jgs.13153 PMC4270076

[29] Dean T, Koné A, Martin L et al (2024) Understanding the extent of polypharmacy and its association with health service utilization among persons with cancer and multimorbidity: a population-based retrospective cohort study in Ontario, Canada. J Pharm Pract 37(1):35–46. 10.1177/08971900221117105 35861340 10.1177/08971900221117105 PMC10804697

[30] Doumat G, Daher D, Itani M et al (2023) The effect of polypharmacy on healthcare services utilization in older adults with comorbidities: a retrospective cohort study. BMC Prim Care 24(1):120. 10.1186/s12875-023-02070-0 37237338 10.1186/s12875-023-02070-0 PMC10214698

[31] Barrio-Cortes J, Benito-Sánchez B, Villimar-Rodriguez AI et al (2023) Differences in healthcare service utilization in patients with polypharmacy according to their risk level by adjusted morbidity groups: a population-based cross-sectional study. J Pharm Policy Pract 16(1):161. 10.1186/s40545-023-00665-7 38017572 10.1186/s40545-023-00665-7 PMC10683272

[32] Australian Institute of Health and Welfare (AIHW). Rural and remote health 2023 [Available from: https://www.aihw.gov.au/reports/rural-remote-australians/rural-and-remote-health].

[33] Wakerman J, Humphreys JS (2019) Better health in the bush”: why we urgently need a national rural and remote health strategy. Med J Aust 210(5):202–3.e1. 10.5694/mja2.50041 30801723 10.5694/mja2.50041

[34] Almaaitah S, Ciemins EL, Joshi V et al (2020) Variation in patient smoking cessation rates among health-care providers: an observational study. Chest 158(5):2038–46. 10.1016/j.chest.2020.05.599 32561440 10.1016/j.chest.2020.05.599

[35] Bailey SR, Stevens VJ, Fortmann SP et al (2018) Long-term outcomes from repeated smoking cessation assistance in routine primary care. Am J Health Promot 32(7):1582–90. 10.1177/0890117118761886 29534598 10.1177/0890117118761886 PMC6342013

[36] Taylor GMJ, Dalili MN, Semwal M et al (2017) Internet-based interventions for smoking cessation. Cochrane Database Syst Rev 9(9):Cd007078. 10.1002/14651858.CD007078.pub5 28869775 10.1002/14651858.CD007078.pub5 PMC6703145

[37] Villar A. Response bias. In Encyclopedia of Survey Research Methods 2008 [Available from: https://methods.sagepub.com/ency/edvol/encyclopedia-of-survey-research-methods/chpt/response-bias].

[38] Australian Institute of Health and Welfare (AIHW). Medicare-subsidised GP, allied health and specialist health care across local areas: 2022–23 2024 [Available from: https://www.aihw.gov.au/reports/primary-health-care/medicare-subsidised-care-2022-23/contents/technical-notes/summary].

[39] Farrington N, Richardson A, Bridges J (2020) Interventions for older people having cancer treatment: a scoping review. J Geriatr Oncol 11(5):769–783. 10.1016/j.jgo.2019.09.015 31699674 10.1016/j.jgo.2019.09.015

