# Supplementary material for: Rates of medical service utilisation in people with and without cancer: an Australian cohort study
Source: Support Care Cancer. 2026 Mar 11;34(4):305. doi: 10.1007/s00520-026-10534-z (PMC12979285; doi:10.1007/s00520-026-10534-z)
Supplement: Supplementary file 1 — (DOCX 77.3 KB) [file 520_2026_10534_MOESM1_ESM.docx]

**Supplementary Table 1** Patterns of medical service use identified using latent class analysis in people with and without cancer and their characteristics

|  | **Cancer, N=3636** | | | **Non-cancer, N=18,477** | | |
| --- | --- | --- | --- | --- | --- | --- |
| **Characteristics** | **Pattern 1**  Low user  n=971 | **Pattern 2**  Predominantly pathology tests  n=1618 | **Pattern 3**  High user  n=1047 | **Pattern 1**  Low user  n=7068 | **Pattern 2**  Predominantly pathology tests  n=8166 | **Pattern 3**  High user  n=3243 |
| **Sex**  Female | 511 (53) | 942 (58) | 582 (56) | 3151 (45) | 4887 (60) | 2009 (62) |
| Male | 460 (47) | 676 (42) | 465 (44) | 3917 (55) | 3279 (40) | 1234 (38) |
| **Age group**  <65 years | 601 (62) | 784 (48) | 315 (30) | 6346 (90) | 6501 (80) | 1869 (58) |
| ≥65 years | 370 (38) | 834 (52) | 732 (70) | 722 (10) | 1665 (20) | 1374 (42) |
| **Country of birth**  Australia | 782 (81) | 1244 (77) | 805 (77) | 4855 (69) | 5268 (65) | 2195 (68) |
| Others | 189 (19) | 374 (23) | 242 (23) | 2213 (31) | 2898 (35) | 1048 (32) |
| **Geographical location**  Major cities | 543 (56) | 961 (59) | 699 (67) | 4580 (65) | 5433 (67) | 2154 (66) |
| Inner/ outer regionals/ remote | 428 (44) | 657 (41) | 348 (33) | 2488 (35) | 2733 (33) | 1089 (34) |
| **Marital status**  Married | 502 (52) | 894 (55) | 623 (60) | 3608 (51) | 4473 (55) | 1849 (57) |
| Never married | 149 (15) | 212 (13) | 84 (8) | 2176 (31) | 1814 (22) | 462 (14) |
| Separated/ widowed/ divorced | 320 (33) | 512 (32) | 340 (32) | 1284 (18) | 1879 (23) | 932 (29) |
| **Education level**  Postgraduate | 104 (11) | 189 (12) | 100 (10) | 932 (13) | 992 (12) | 375 (12) |
| Bachelor | 161 (17) | 286 (18) | 182 (17) | 1617 (23) | 1855 (23) | 598 (18) |
| Diploma | 131 (13) | 176 (11) | 125 (12) | 777 (11) | 976 (12) | 362 (11) |
| Certificate | 227 (23) | 323 (20) | 182 (17) | 1534 (22) | 1558 (19) | 582 (18) |
| No non-school qualification/not known | 348 (36) | 644 (40) | 458 (44) | 2208 (31) | 2785 (34) | 1326 (41) |
| **Employment status**  Employed | 516 (53) | 668 (41) | 295 (28) | 5365 (76) | 5322 (65) | 1542 (48) |
| Unemployed/not in labour force | 455 (47) | 950 (59) | 752 (72) | 1703 (24) | 2844 (35) | 1701 (52) |
| **Equivalised personal weekly income**  Decile 1-2 (most disadvantaged) | 88 (9) | 181 (11) | 92 (9) | 787 (11) | 987 (12) | 375 (12) |
| Decile 3-4 | 211 (22) | 443 (27) | 346 (33) | 868 (12) | 1483 (18) | 890 (27) |
| Decile 5-6 | 195 (20) | 341 (21) | 234 (22) | 1232 (17) | 1583 (19) | 587 (18) |
| Decile 7-8 | 206 (21) | 251 (16) | 159 (15) | 1637 (23) | 1715 (21) | 540 (17) |
| Decile 9-10 | 199 (21) | 282 (17) | 137 (13) | 2013 (29) | 1827 (22) | 574 (18) |
| Not known | 72 (7) | 120 (7) | 79 (8) | 531 (8) | 571 (7) | 277 (8) |
| **Body mass index**  Normal/underweight/missing | 350 (36) | 529 (33) | 326 (31) | 2678 (38) | 3007 (37) | 1093 (34) |
| Overweight | 359 (37) | 583 (36) | 400 (38) | 2709 (38) | 2775 (34) | 1130 (35) |
| Obese | 262 (27) | 506 (31) | 321 (31) | 1681 (24) | 2384 (29) | 1020 (32) |
| **Smoking status**  Never smoked | 430 (44) | 802 (50) | 543 (52) | 3734 (53) | 4472 (55) | 1772 (55) |
| Ex-smoker | 389 (40) | 671 (41) | 459 (44) | 2094 (30) | 2675 (33) | 1222 (38) |
| Current smoker | 152 (16) | 145 (9) | 45 (4) | 1240 (17) | 1019 (12) | 249 (8) |
| **Met recommended vegetable/ fruits guidelines**  Met both | 63 (7) | 138 (9) | 85 (8) | 362 (5) | 509 (6) | 240 (7) |
| Met either | 447 (46) | 808 (50) | 547 (52) | 2980 (42) | 3664 (45) | 1596 (49) |
| Not met/ missing | 461 (47) | 672 (41) | 415 (40) | 3726 (53) | 3993 (49) | 1407 (43) |
| **Alcohol intake**  Not applicable/ not known | 165 (17) | 331 (20) | 240 (23) | 1221 (17) | 1846 (23) | 752 (23) |
| Everyday | 104 (11) | 156 (10) | 113 (11) | 408 (6) | 428 (5) | 231 (7) |
| 2-6 days a week | 443 (46) | 666 (41) | 409 (39) | 3194 (45) | 3159 (39) | 1191 (37) |
| 1-3 days a month | 150 (15) | 257 (16) | 140 (13) | 1287 (18) | 1425 (17) | 528 (16) |
| Less than once a month | 109 (11) | 208 (13) | 145 (14) | 958 (14) | 1308 (16) | 541 (17) |
| **Met physical activity guidelines**  Yes | 228 (24) | 424 (26) | 254 (24) | 1356 (19) | 1683 (21) | 700 (22) |
| No | 743 (76) | 1194 (74) | 793 (76) | 5712 (81) | 6483 (79) | 2543 (78) |
| **Number of health conditions (excluding cancer)**  0 | 163 (17) | 160 (10) | 75 (7) | 2267 (32) | 1685 (21) | 356 (11) |
| 1-2 | 495 (51) | 742 (46) | 372 (36) | 3660 (52) | 4148 (51) | 1417 (44) |
| 3-4 | 252 (26) | 542 (33) | 427 (41) | 998 (14) | 1878 (23) | 1098 (34) |
| ≥5 | 61 (6) | 174 (11) | 173 (16) | 143 (2) | 455 (6) | 372 (11) |
| **Polypharmacy**  No | 890 (92) | 1361 (84) | 720 (69) | 6930 (98) | 7605 (93) | 2567 (79) |
| Yes | 81 (8) | 257 (16) | 327 (31) | 138 (2) | 561 (7) | 676 (21) |
| **Cancer status**  Current cancer | 85 (9) | 221 (14) | 228 (22) | N/A | N/A | N/A |
| Non-current | 886 (91) | 1397 (86) | 819 (78) |  |  |  |

**Supplementary Table 2** Patterns of medical service use identified using latent class analysis by cancer and age group and their characteristics

|  | **Cancer, younger**  **N=1700** | | | **Non-cancer, younger**  **N=14,716** | | | **Cancer, older**  **N=1936** | | | **Non-cancer, older**  **N=3761** | | |
| --- | --- | --- | --- | --- | --- | --- | --- | --- | --- | --- | --- | --- |
| **Characteristics** | **Pattern 1**  Low user  n=587  (34%) | **Pattern 2**  Pre-dominantly pathology tests  n=643  (38%) | **Pattern 3**  High user  n=470  (28%) | **Pattern 1**  Low user  n=6336  (43%) | **Pattern 2**  Pre-dominantly pathology tests  n=6453  (44%) | **Pattern 3**  High user  n=1927  (13%) | **Pattern 1**  Low user  n=424  (22%) | **Pattern 2**  Pre-dominantly pathology tests  n=837  (43%) | **Pattern 3**  High user  n=675  (35%) | **Pattern 1**  Low user  n=1019  (27%) | **Pattern 2**  Pre-dominantly pathology tests  n=1552  (41%) | **Pattern 3**  High user  n=1190  (32%) |
| **Sex**  Female | 310 (53) | 424 (66) | 295 (63) | 2764 (44) | 3915 (61) | 1273 (66) | 228 (54) | 432 (52) | 346 (51) | 527 (52) | 896 (58) | 672 (57) |
| Male | 277 (47) | 219 (34) | 175 (37) | 3572 (56) | 2538 (39) | 654 (34) | 196 (46) | 405 (48) | 329 (49) | 492 (48) | 656 (42) | 518 (44) |
| **Country of birth**  Australia | 497 (85) | 516 (80) | 387 (82) | 4391 (69) | 4257 (66) | 1361 (71) | 315 (74) | 608 (73) | 508 (75) | 636 (62) | 914 (59) | 759 (64) |
| Others | 90 (15) | 127 (20) | 83 (18) | 1945 (31) | 2196 (34) | 566 (29) | 109 (26) | 229 (27) | 167 (25) | 383 (38) | 638 (41) | 431 (36) |
| **Geographical location**  Major cities | 331 (56) | 394 (61) | 310 (66) | 4137 (65) | 4437 (69) | 1330 (69) | 232 (55) | 486 (58) | 450 (67) | 602 (59) | 903 (58) | 758 (64) |
| Inner/ outer regionals/ remote | 256 (44) | 249 (39) | 160 (34) | 2199 (35) | 2016 (31) | 597 (31) | 192 (45) | 351 (42) | 225 (33) | 417 (41) | 649 (42) | 432 (36) |
| **Marital status**  Married | 303 (52) | 354 (55) | 270 (57) | 3250 (51) | 3547 (55) | 1118 (58) | 221 (52) | 465 (56) | 406 (60) | 531 (52) | 815 (53) | 669 (56) |
| Never married | 128 (22) | 125 (19) | 91 (19) | 2121 (34) | 1694 (26) | 415 (22) | 22 (5) | 50 (6) | 29 (4) | 65 (6) | 108 (7) | 49 (4) |
| Separated/ widowed/ divorced | 156 (27) | 164 (26) | 109 (23) | 965 (15) | 1212 (19) | 394 (20) | 181 (43) | 322 (39) | 240 (36) | 423 (42) | 629 (40) | 472 (40) |
| **Education level**  Postgraduate | 79 (13) | 94 (15) | 74 (16) | 869 (14) | 894 (14) | 278 (14) | 27 (6) | 72 (9) | 47 (7) | 80 (8) | 86 (6) | 92 (8) |
| Bachelor | 101 (17) | 118 (18) | 98 (21) | 1535 (24) | 1633 (25) | 451 (23) | 69 (16) | 139 (17) | 104 (15) | 120 (12) | 189 (12) | 142 (12) |
| Diploma | 82 (14) | 85 (13) | 59 (13) | 717 (11) | 833 (13) | 261 (14) | 49 (12) | 81 (10) | 76 (11) | 81 (8) | 127 (8) | 96 (8) |
| Certificate | 157 (27) | 136 (21) | 92 (20) | 1389 (22) | 1262 (20) | 373 (19) | 75 (18) | 159 (19) | 113 (17) | 195 (19) | 260 (17) | 195 (16) |
| No non-school qualification/ not known | 168 (29) | 210 (33) | 147 (31) | 1826 (29) | 1831 (28) | 564 (29) | 204 (48) | 386 (46) | 335 (50) | 543 (53) | 890 (57) | 665 (56) |
| Not known |  |  |  |  |  |  |  |  |  |  |  |  |
| **Employment status**  Employed | 452 (77) | 451 (70) | 305 (65) | 5204 (82) | 5007 (78) | 1379 (72) | 59 (14) | 127 (15) | 85 (13) | 215 (21) | 241 (16) | 183 (15) |
| Unemployed/not in labour force | 135 (23) | 192 (30) | 165 (35) | 1132 (18) | 1446 (22) | 548 (28) | 365 (86) | 710 (85) | 590 (87) | 804 (79) | 1311 (84) | 1007 (85) |
| **Equivalised personal weekly income**  Decile 1-2 (most disadvantaged) | 55 (9) | 89 (14) | 69 (15) | 714 (11) | 822 (13) | 263 (14) | 39 (9) | 67 (8) | 42 (6) | 99 (10) | 141 (9) | 110 (9) |
| Decile 3-4 | 75 (13) | 98 (15) | 71 (15) | 603 (10) | 722 (11) | 281 (15) | 153 (36) | 329 (39) | 274 (41) | 388 (38) | 729 (47) | 518 (44) |
| Decile 5-6 | 105 (18) | 99 (15) | 77 (16) | 1067 (17) | 1215 (19) | 317 (16) | 101 (24) | 218 (26) | 170 (25) | 234 (23) | 322 (21) | 247 (21) |
| Decile 7-8 | 136 (23) | 136 (21) | 105 (22) | 1550 (25) | 1538 (24) | 435 (23) | 67 (16) | 90 (11) | 82 (12) | 111 (11) | 148 (9) | 110 (9) |
| Decile 9-10 | 175 (30) | 172 (27) | 122 (26) | 1942 (31) | 1727 (27) | 504 (26) | 29 (7) | 63 (8) | 57 (8) | 89 (9) | 76 (5) | 76 (6) |
| Not known | 41 (7) | 49 (8) | 26 (6) | 460 (7) | 429 (7) | 127 (7) | 35 (8) | 70 (8) | 50 (7) | 98 (10) | 136 (9) | 129 (11) |
| **Body mass index**  Normal/underweight/missing | 204 (35) | 220 (34) | 158 (34) | 2418 (38) | 2459 (38) | 696 (36) | 163 (38) | 251 (30) | 209 (31) | 353 (35) | 491 (32) | 361 (30) |
| Overweight | 219 (27) | 213 (33) | 155 (33) | 2425 (38) | 2132 (33) | 606 (31) | 153 (36) | 328 (39) | 274 (41) | 385 (38) | 582 (37) | 484 (41) |
| Obese | 164 (28) | 210 (33) | 157 (33) | 1493 (24) | 1862 (29) | 625 (32) | 108 (26) | 258 (31) | 192 (28) | 281 (28) | 479 (31) | 345 (29) |
| **Smoking status**  Never smoked | 244 (42) | 310 (48) | 241 (51) | 3352 (53) | 3591 (56) | 1077 (56) | 208 (49) | 422 (50) | 350 (52) | 522 (51) | 809 (52) | 627 (53) |
| Ex-smoker | 224 (38) | 252 (39) | 188 (40) | 1813 (29) | 1963 (30) | 648 (34) | 182 (43) | 368 (44) | 305 (45) | 412 (40) | 639 (41) | 516 (43) |
| Current smoker | 119 (20) | 81 (13) | 41 (9) | 1171 (18) | 899 (14) | 202 (10) | 34 (8) | 47 (6) | 20 (3) | 85 (8) | 104 (7) | 47 (4) |
| **Met recommended vegetable/ fruits guidelines**  Met both | 31 (5) | 48 (8) | 37 (8) | 312 (5) | 365 (6) | 141 (7) | 42 (10) | 77 (9) | 51 (8) | 67 (7) | 141 (9) | 85 (7) |
| Met either | 246 (42) | 291 (45) | 218 (46) | 2584 (41) | 2811 (44) | 862 (45) | 225 (53) | 450 (54) | 372 (55) | 535 (52) | 779 (50) | 669 (56) |
| Not met/ missing | 310 (53) | 304 (47) | 215 (46) | 3440 (54) | 3277 (51) | 924 (48) | 157 (37) | 310 (37) | 252 (37) | 417 (41) | 632 (41) | 436 (37) |
| **Alcohol intake**  Not applicable/ not known | 79 (13) | 115 (18) | 83 (18) | 1026 (16) | 1350 (21) | 398 (21) | 96 (23) | 196 (23) | 167 (25) | 282 (28) | 449 (29) | 314 (26) |
| Everyday | 45 (8) | 39 (6) | 32 (7) | 318 (5) | 239 (4) | 84 (4) | 61 (14) | 108 (13) | 88 (13) | 129 (13) | 165 (11) | 132 (11) |
| 2-6 days a week | 294 (50) | 282 (44) | 202 (43) | 2927 (46) | 2567 (40) | 749 (39) | 165 (39) | 319 (38) | 256 (38) | 369 (36) | 528 (34) | 404 (34) |
| 1-3 days a month | 103 (18) | 124 (19) | 75 (16) | 1203 (19) | 1234 (19) | 363 (19) | 56 (13) | 104 (12) | 85 (13) | 104 (10) | 189 (12) | 147 (12) |
| Less than once a month | 66 (11) | 83 (13) | 78 (17) | 862 (14) | 1063 (16) | 333 (17) | 46 (11) | 110 (13) | 79 (12) | 135 (13) | 221 (14) | 193 (16) |
| **Met physical activity guidelines**  Yes | 125 (21) | 125 (19) | 75 (16) | 1147 (18) | 1178 (18) | 319 (17) | 120 (28) | 279 (33) | 182 (27) | 303 (30) | 449 (29) | 343 (29) |
| No | 462 (79) | 518 (81) | 395 (84) | 5189 (82) | 5275 (82) | 1608 (83) | 304 (72) | 558 (67) | 493 (73) | 716 (70) | 1103 (71) | 847 (71) |
| **Number of health conditions (excluding cancer)**  0 | 121 (21) | 87 (13) | 51 (11) | 2140 (34) | 1502 (23) | 288 (15) | 47 (11) | 51 (6) | 41 (6) | 164 (16) | 142 (9) | 72 (6) |
| 1-2 | 322 (55) | 306 (48) | 185 (39) | 3293 (52) | 3336 (52) | 905 (47) | 191 (45) | 369 (44) | 236 (35) | 516 (51) | 715 (46) | 460 (39) |
| 3-4 | 119 (20) | 181 (28) | 167 (36) | 796 (13) | 1313 (20) | 560 (29) | 148 (35) | 318 (38) | 288 (43) | 284 (28) | 539 (35) | 482 (41) |
| ≥5 | 25 (4) | 69 (11) | 67 (14) | 107 (2) | 302 (5) | 174 (9) | 38 (9) | 99 (12) | 110 (16) | 55 (5) | 156 (10) | 176 (15) |
| **Polypharmacy**  No | 574 (98) | 591 (92) | 401 (85) | 6284 (99) | 6253 (97) | 1719 (89) | 345 (81) | 642 (77) | 418 (62) | 892 (88) | 1174 (76) | 780 (65) |
| Yes | 13 (2) | 52 (8) | 69 (15) | 52 (1) | 200 (3) | 208 (11) | 79 (19) | 195 (23) | 257 (38) | 127 (12) | 378 (24) | 410 (35) |
| **Cancer status**  Current cancer | 43 (7) | 76 (12) | 101 (22) | N/A | N/A | N/A | 46 (11) | 127 (15) | 141 (21) | N/A | N/A | N/A |
| Non-current | 544 (93) | 567 (88) | 369 (78) |  |  |  | 378 (89) | 710 (85) | 534 (79) |  |  |  |

**Supplementary Figure 1** Rates of medical service use by cancer and age groups

Key: MBS, Medicare Benefits Schedule. The results for ‘any medical services’ are presented in the right square box with a separate y-axis. Crude rate was calculated using the formula: (total number of medical services for each broad types of services by cancer and age group/ total person years of follow-up by cancer and age group) X 100.

**Supplementary Figure 2** The prevalence distribution of the demographic characteristics by cancer and age group and patterns of medical service use identified using latent class analysis

| **Cancer, younger** | **Non-cancer, younger** |
| --- | --- |
|  |  |
| **Cancer, older** | **Non-cancer, older** |
|  |  |

The percentage (%) of the characteristics in each pattern is shown

**Supplementary Figure 3** The prevalence distribution of the lifestyle and other conditions by cancer and age group and patterns of medical service use identified using latent class analysis

| **Cancer, younger** | **Non-cancer, younger** |
| --- | --- |
|  |  |
| **Cancer, older** | **Non-cancer, older** |
|  |  |

The percentage (%) of the characteristics in each pattern is shown
